# Beneficial changes on plasma apolipoproteins A and B, high density lipoproteins and oxidized low density lipoproteins in obese women after bariatric surgery: comparison between gastric bypass and sleeve gastrectomy

**DOI:** 10.1186/s12944-018-0794-5

**Published:** 2018-06-20

**Authors:** J. M. Gómez-Martin, J. A. Balsa, E. Aracil, M. Cuadrado-Ayuso, M. Rosillo, G. De la Peña, M. A. Lasunción, H. F. Escobar-Morreale, J. I. Botella-Carretero

**Affiliations:** 1 Department of Endocrinology and Nutrition, Hospital Universitario Ramón y Cajal & Instituto Ramón y Cajal de Investigación Sanitaria (IRYCIS), Carretera de Colmenar Km. 9.1, 28034 Madrid, Spain; 2 Department of Vascular Surgery, Hospital Universitario Ramón y Cajal & Instituto Ramón y Cajal de Investigación Sanitaria (IRYCIS), 28034 Madrid, Spain; 3 Department of General and Gastrointestinal Surgery, Hospital Universitario Ramón y Cajal & Instituto Ramón y Cajal de Investigación Sanitaria (IRYCIS), 28034 Madrid, Spain; 4 Department of Clinical Biochemistry, Hospital Universitario Ramón y Cajal & Instituto Ramón y Cajal de Investigación Sanitaria (IRYCIS), 28034 Madrid, Spain; 5 Department of Biochemistry Research, Hospital Universitario Ramón y Cajal & Instituto Ramón y Cajal de Investigación Sanitaria (IRYCIS), 28034 Madrid, Spain; 6 Universidad de Alcalá & Centro de Investigación Biomédica en Red Diabetes y Enfermedades Metabólicas Asociadas (CIBERDEM), Madrid, Spain; 7 Centro de Investigación Biomédica en Red Fisiopatología de la Obesidad y Nutrición (CIBEROBN), Madrid, Spain; 8 Department of Endocrinology and Nutrition, Hospital Infanta Sofía & Universidad Europea, Madrid, Spain

**Keywords:** Obesity, Bariatric surgery, Lipid metabolism, Low density lipoproteins, Oxidized LDL, Metabolic syndrome

## Abstract

**Background:**

The beneficial effects in lipid profiles after obesity surgery might be associated with the decrease in cardiovascular risk. However, direct comparison between different surgical techniques has not been extensively performed.

**Methods:**

In the present study we compare 20 obese women submitted to laparoscopic Roux en Y gastric bypass (RYGB) with 20 women submitted to sleeve gastrectomy (SG). Twenty control women matched for age and baseline cardiovascular risk were also included. Both patients and controls were followed up for 1 year after surgery or conventional treatment with diet and exercise, respectively. Lipid profiles were measured at baseline, 6 and 12 months later. Carotid intima-media thickness was measured by ultrasonography at baseline and at the end of the study.

**Results:**

Women submitted to bariatric surgery showed a decrease in total cholesterol, triglycerides, oxidized-LDL and ApoB, and an increase in HDL and ApoA concentrations that occurred regardless of the surgical procedure. LDL concentrations, however, decreased only after RYGB whereas Lp(a) showed no changes. We did not observe any correlation between the changes in serum lipid concentrations and those in carotid intima-media thickness.

**Conclusions:**

Sleeve gastrectomy and gastric bypass induce a similar beneficial effect on serum lipids in women with high cardiovascular risk 1 year after surgery.

## Background

Obesity, a major public health issue [1] is associated with an increase in mortality and many comorbidities [2, 3]. The latter include several cardiovascular risk factors such as type 2 diabetes, dyslipidemia, hypertension, and prothrombotic states, among others [4, 5].

The use of obesity surgery procedures has increased steadily in the past decades because such procedures characteristically result in much larger long-term weight loss than that usually achieved following diet and life-style modification [6]. This tendency has also been driven by the low complications of modern laparoscopic surgical procedures that, although not free of nutritional and metabolic issues [7–10], clearly compensate for the substantial long-term morbidity and mortality of severe obesity [11]. The remission of many metabolic and hormonal disorders [12–16] and the reduction in cardiovascular risk are also superior with obesity surgery [17, 18].

Focusing on the pro-atherogenic lipid profile, weight loss of at least 5% may result in a decrease in all apolipoprotein B (ApoB)-containing lipoproteins and an increase in the circulating concentrations of large high density-lipoprotein (HDL) particles [19]. Besides, the marked weight loss that usually follows bariatric surgery also ameliorates the atherogenicity of plasma lipoproteins by reducing the ApoB-containing lipoproteins and oxidised low density-lipoproteins (oxLDL), as well as by increasing the HDL-2 subfraction [20].

We have recently reported that both Roux-en-Y gastric bypass (RYGB) and sleeve gastrectomy (SG) induced a decrease in carotid intima-media thickness that was superior to that attained after medical treatment [21]. We also found an increase in HDL concentrations after bariatric surgery regardless of the surgical technique applied, whereas LDL levels only decreased after RYGB. Although the latter might be considered as an advantage of RYGB over SG in terms of decreasing cardiovascular risk factors, an expanded analysis in lipid profiles are necessary before reaching such a conclusion. Therefore, in the present study we report an expanded analysis of circulating lipids after both RYGB and SG. As it will be seen, the beneficial effects observed on circulating HDL, apolipoprotein A1 (ApoA1), ApoB and oxLDL were of similar magnitude after both surgical techniques.

## Methods

### Patients and study design

We included 40 women submitted to obesity surgery who met the criteria for the metabolic syndrome [22] and, hence, had a high cardiovascular risk. The Systematic Coronary Risk Evaluation (SCORE) – a validated and recommended method for estimating cardiovascular risk in Spanish population [23] – was also calculated for each patient and control at baseline. Half of them were submitted to laparoscopic RYGB and half were submitted to SG following international guidelines for obesity surgery and our hospital’s own protocol. This local protocol allocates patients with body mass index (BMI) above 45 Kg/m^2^ preferentially to RYGB - although patients with BMI > 45 Kg/m^2^ were not excluded for a SG procedure - and, therefore, it precluded randomization of the subjects for the surgical technique finally applied. Other than BMI, no other characteristic of the patients influenced the allocation to RYGB or SG. Previous diagnosis of type 2 diabetes mellitus and hypertension followed current international standards [24, 25].

The main characteristics of RYGB procedure include a 20–40 ml gastric pouch, a biliopancreatic limb measuring 80–100 cm from Treitz ligament, and a 120–200 cm-long alimentary limb. SG was performed with a laparoscopic linear stapler calibrated with a 32F orogastric tube. Twenty control women matched for age and cardiovascular risk were also recruited and submitted to treatment with diet and life-style modification.

Exclusion criteria included mental impairment, uncontrolled psychiatric conditions or active substance abuse, active neoplastic disease, pregnancy, unstable or incurable serious pre-existing comorbidities, and current treatment with thiazolidinediones. Both patients and controls were evaluated at baseline and 1 year after surgery or after starting conventional treatment with diet and life-style modification, respectively. Patients were also revaluated 6 months after surgery for anthropometric and analytical evaluation (but not for carotid intima-media measurements). Data of the change in carotid intima-media thickness of these patients were reported earlier [21].

Basal blood samples were obtained in every woman after an overnight fast. Office blood pressure, waist circumference (WC), weight and height were also recorded and BMI calculated. Excess body weight (EBW) was calculated as baseline body weight minus the ideal weight [26, 27]. Excess weight loss (EWL) was calculated as the percentage of weight loss from baseline EBW.

Control women were prescribed a modified Mediterranean diet, aimed to a caloric restriction of 400–500 kcal per day, by an expert dietitian. Patients attended bimonthly interviews with the dietitian for nutritional counseling and follow-up during the study period. For those women submitted to obesity surgery, a 1400 kcal per day preoperative diet was prescribed two to 3 months before the intervention and, during the first month after surgery, a transition liquid to solid diet was supervised by a dietitian. A fractionated diet with 5 to 6 mixed meals was then prescribed throughout the study period.

### Assays

Levels of fasting HDL cholesterol were measured in supernatant after plasma precipitation with phosphotungstic acid and Mg^2+^ (Boehringer Mannheim GmbH, Mannheim, Germany). Levels of total cholesterol and triglycerides were measured by enzymatic methods (Menarini Diagnostica, Florence, Italy). The LDL cholesterol level was calculated by using Friedewald’s formula. OxLDL was measured in duplicate by enzyme link immunosorbent assay (ELISA) in fresh EDTA plasma samples using a commercial kit (Mercodia Oxidized LDL ELISA, Mercodia Uppsala, Sweden) with a detection limit of 0.6 mg/dl, and an intra- and interassay CVs of 6.3 and 4.7% respectively. Serum lipoprotein a [Lp(a)], apolipoprotein A1 (ApoA1), and ApoB levels were measured by standard colorimetric methods, using the Architect ci8200 analyzer (Abbot Diagnostics, Berkshire, UK). The Lp (a) assay had a detection limit of 0.83 mg/dl, an intrassay CV of 0.7% and an interassay CVs of 1.9%. Intra- and interassay CVs were 1.4 and 0.7% for ApoA1 and 2.3 and 1.5% for ApoB, respectively. Detection limits for both apolipoproteins were < 3 mg/dl.

### Statistics

A priori power analysis was performed as reported before [21]. Results are expressed as means ± SD unless otherwise stated. The Kolmogorov–Smirnov statistic was used to assess normality. Logarithmic or square root transformations were applied if needed. One-way analysis of variance followed by Dunnet’s or Tuckey’s tests was used to compare the central tendencies of the different groups. For non-parametric variables Kruskal-Wallis test followed by Mann-Whitney U tests were employed. For discontinuous variables we used the χ^2^ test and Fisher’s exact test. Comparisons of continuous variables before and after surgery were performed using repeated-measures general linear model (GLM) analysis, and the group of subjects (controls, RYGB or SG) was introduced as the between-subjects effect. The comparison of the magnitude of the changes in the dependent variables during follow up (calculated as the percentage increment from baseline) were performed by one-way analysis of variance (ANOVA) followed by *posthoc* analyses (Dunnet’s and Tuckey’s tests) or by Kruskal-Wallis and Mann-Whitney U tests as appropriate. Bivariate correlation was employed to study the association between two continuous variables using Pearson’s tests. Analyses were performed using SPSS 17 (SPSS Inc., Chicago, Illinois). *P* < 0.05 was considered statistically significant.

## Results

The baseline characteristics of the patients are summarized in Table 1. As expected from our surgical protocol, patients submitted to RYGB presented with increased BMI, EBW, and WC (Table 1). We did not observe differences in lipid profiles, blood pressure or percentage of smokers between the groups (Table 1). On the other hand, the number of patients on statins were higher in the group submitted to RYGB than in the groups treated with SG diet and life-style modification (Table 1). The cardiovascular risk measured by the SCORE was high (5–9% at 10 years) in all patients except for one control, one patient submitted to SG and one patient submitted to RYGB, all of whom showed very high risk (> 10% at 10 years).

**Table 1:** Baseline characteristics of the included women (*n* = 58)

	Controls (*n* = 18)	SG (*n* = 20)	RYGB (*n* = 20)
Age (years)	52 ± 7	46 ± 9	48 ± 8
Body mass index (kg/m^2^)	42 ± 6	43 ± 4	47 ± 6*†
Excess body weight (kg)	42 ± 15	45 ± 11	57 ± 17*†
Waist circumference (cm)	118 ± 13	119 ± 9	133 ± 13*†
Systolic blood pressure (mmHg)	146 ± 20	146 ± 24	139 ± 19
Diastolic blood pressure (mmHg)	92 ± 15	87 ± 13	87 ± 12
Total cholesterol (mg/dL)	204 ± 34	210 ± 51	201 ± 30
HDL-cholesterol (mg/dL)	48 ± 11	49 ± 14	47 ± 7
LDL-cholesterol (mg/dL)	129 ± 28	132 ± 44	125 ± 26
oxLDL (mg/dL)	53 ± 7	54 ± 16	59 ± 12
ApoA1 (mg/dL)	159 ± 21	156 ± 28	159 ± 26
ApoB (mg/dL)	105 ± 13	110 ± 25	108 ± 22
Lp(a) (mg/dL)	38 ± 59	43 ± 64	40 ± 39
Triglycerides (mg/dL)	135 ± 51	153 ± 88	136 ± 61
Diabetes mellitus	4 (22%)	5 (25%)	5 (25%)
Oral antidiabetic drugs	4 (22%)	4 (20%)	5 (25%)
Dyslipidemia	8 (44%)	4 (20%)	7 (35%)
Statins	3 (16%)	1 (4%)	7 (35%)*†
Hypertension	9 (50%)	12 (60%)	13 (65%)
Antihypertensive drugs	7 (37%)	12 (60%)	13 (65%)
Smokers	5 (28%)	7 (35%)	7 (35%)

Data are means ± SD or counts (percentage)

*SG* sleeve gastrectomy, *RYGB* Roux in Y gastric bypass, *HDL* high density lipoprotein, *LDL* low density lipoprotein, *oxLDL* oxidized low density lipoprotein, *ApoA1* apolipoprotein A1, *ApoB* apolipoprotein B, *Lp(a)* lipoprotein a

*P < 0.05 vs. controls, †*P* < 0.05 vs. SG

**Fig. 1:**
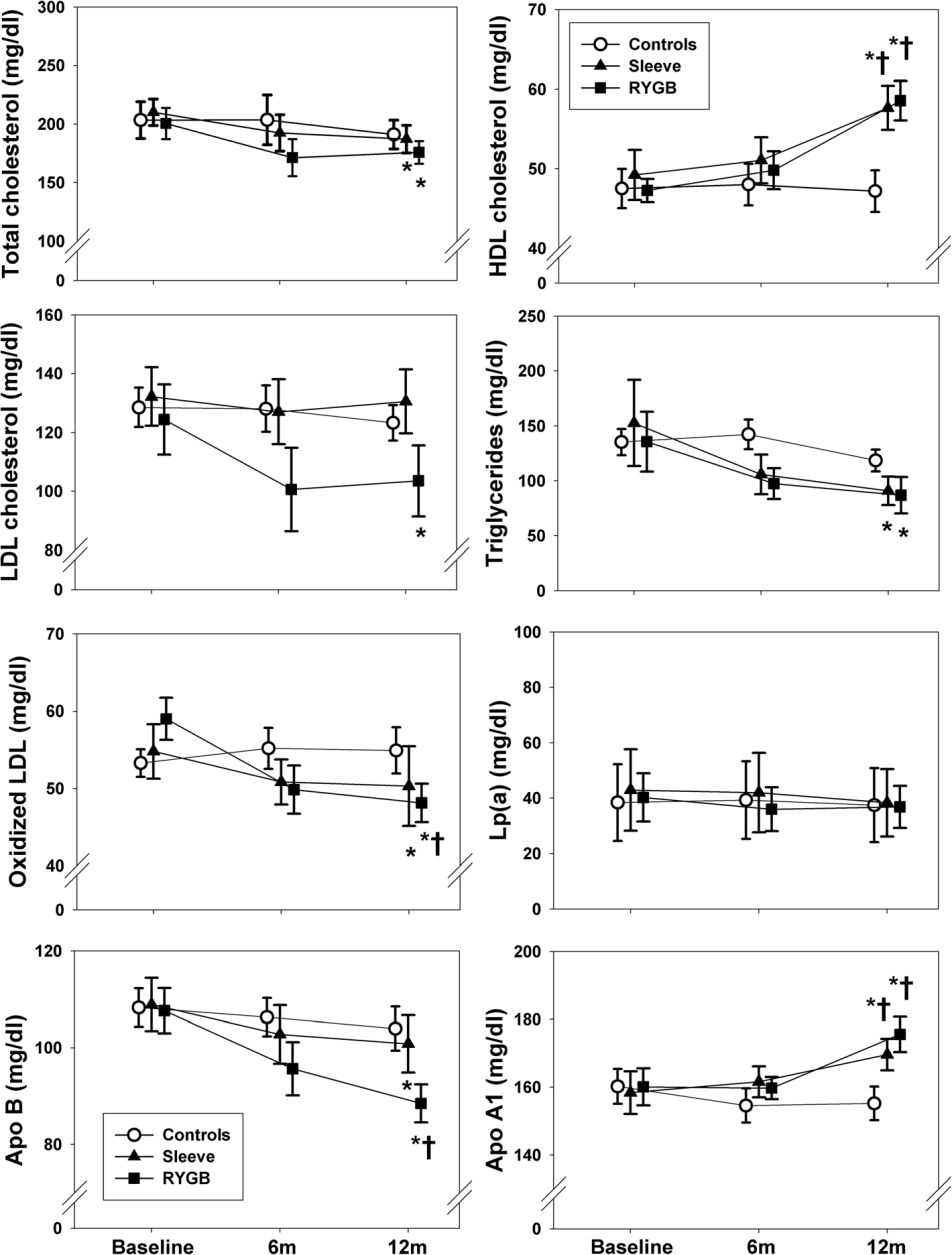
Changes in circulating lipids 1 year after bariatric surgery. Data are expressed as means (circles for controls, triangles for sleeve and squares for RYGB) and SEM (error bars). RYGB: Roux in Y gastric bypass; HDL: high density lipoprotein; LDL: low density lipoprotein; oxLDL: oxidized low density lipoprotein; Lp(a): lipoprotein a; Apo B: apolipoprotein B; Apo A1: apolopoprotein A1. * *P* < 0.05 from baseline, † *P* < 0.05 compared with controls

Compared with non-surgical controls who showed no significant changes in lipid concentrations at the end of the study (Fig. 1), women submitted to bariatric surgery experienced a decrease in total cholesterol, triglycerides, oxLDL and ApoB, and an increase in HDL and ApoA1 concentrations (Fig. 1). The latter changes were similar after sleeve and RYGB, but serum LDL cholesterol decreased only after RYGB (Fig. 1). On the other hand, serum Lp(a) showed no changes in any group (Fig. 1).

Regarding other clinical and analytical variables, a greater EWL and a decrease in BMI, WC and blood pressure were observed after bariatric surgery with no influence of the surgical technique (Table 2). Similarly, carotid intima-media thickness decreased in patients after surgery regardless of the surgical technique but did not change in controls (Table 2). The number of patients on statins was reduced only after RYGB (2, 3 and 3 patients were on statins in the RYGB, SG and control groups at the end of the study, respectively).

**Table 2:** Changes in anthropometric and other variables after 1 year of follow up

	Controls (*n* = 18)	SG (*n* = 20)	RYGB (*n* = 20)
Body mass index (kg/m^2^)	−1.3 ± 4.2	−12.4 ± 3.9*†	−15.9 ± 4.9*†
Excess weight loss (%)	−6.5 ± 19.3	−71.1 ± 23.1*†	−72.4 ± 13.3*†
Waist circumference (cm)	−0.3 ± 4.6	− 16.7 ± 12.4*†	−25.2 ± 13.4*†
Systolic blood pressure (mmHg)	−6.6 ± 18.8	−12.1 ± 23.0*	−17.2 ± 20.0*
Diastolic blood pressure (mmHg)	−6.7 ± 10.1*	−11.2 ± 13.9*	−10.3 ± 17.1*
Fasting glucose (mg/dL)	− 1.4 ± 20.0	−22.7 ± 24.8*†	−26.9 ± 20.4*†
Carotid IMT (mm)	0.01 ± 0.11	−0.11 ± 0.10*†	−0.08 ± 0.09*†

Data are mean differences ± SD

*SG* sleeve gastrectomy, *RYGB* Roux in Y gastric bypass, *IMT* intima-media thickness

*P < 0.05 for the difference from baseline to the end of study, †*P* < 0.05 for the difference vs. controls

**Fig. 2:**
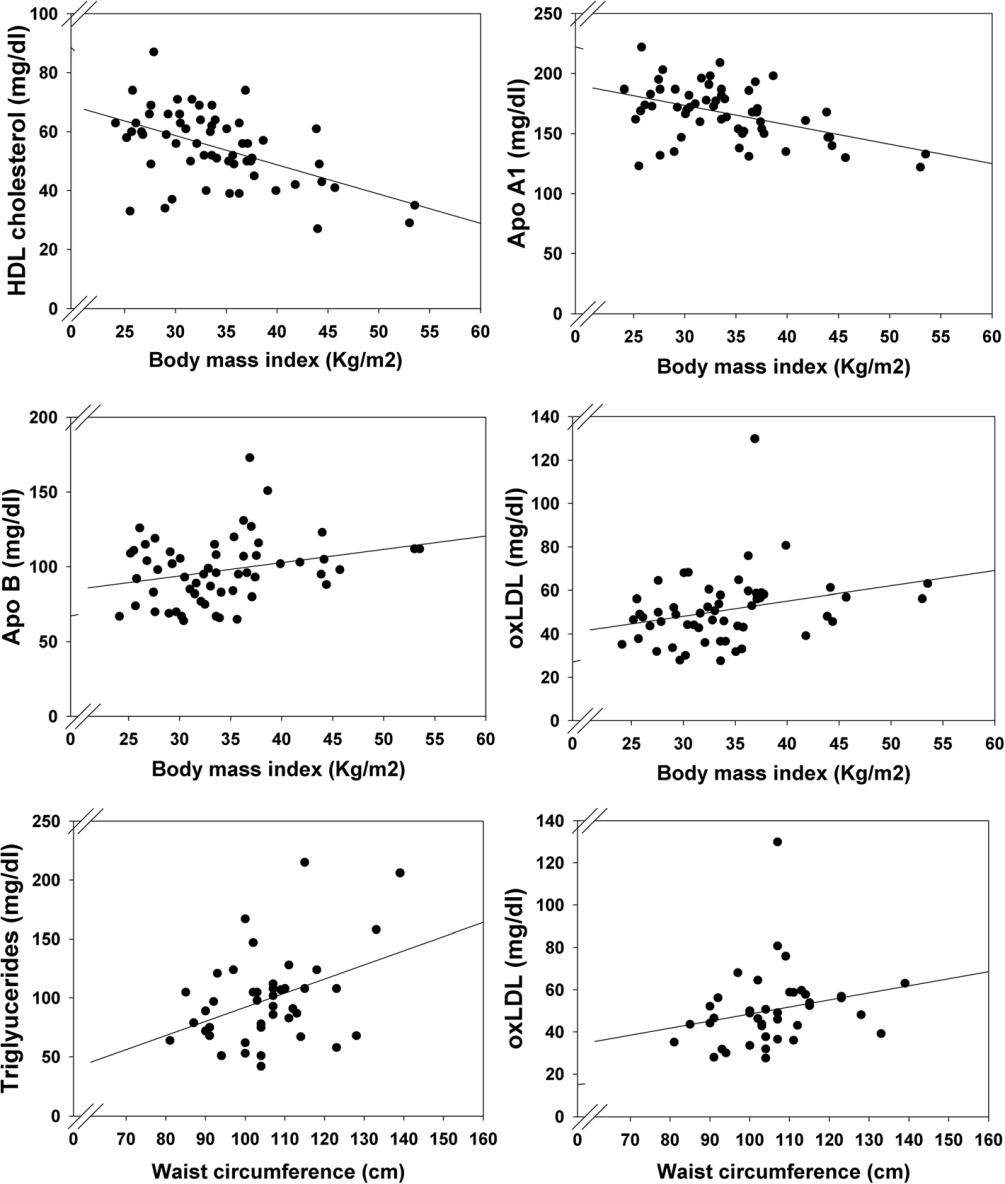
Correlations of body mass index and waist circumference with circulating lipids at the end of the study. Scatterplots show individual data and lines represent the linear regression fit. HDL: high density lipoprotein; oxLDL: oxidized low density lipoprotein; Apo B: apolipoprotein B; Apo A1: apolopoprotein A1. Correlations were all significant at *P* < 0.05

When considering all participants as a whole (both women submitted to surgery and those to diet and exercise), there were no correlations between the changes in carotid intima-media thickness and those observed in any of the lipids analyzed (Table 3). Correlations were no significant either when repeating the analysis separating patients into subgroups of surgery and controls (data not shown). At the end of the study BMI showed a negative correlation with HDL (*r* = − 0.516, *P* < 0.001) and ApoA1 (*r* = − 0.462, *P* < 0.001), and a positive correlation with triglycerides (*r* = 0.499, *P* < 0.001), ApoB (*r* = 0.265, *P* = 0.046) and oxLDL (*r* = 0.277, *P* = 0.040) (Fig. 2). Also, WC showed a positive correlation with triglycerides (*r* = 0.399, *P* = 0.009) and oxLDL (*r* = 0.367, *P* = 0.020) (Fig. 2).

**Table 3:** Correlations of changes in carotid IMT with changes in serum lipid concentrations

	Δ Carotid IMT	
	r	*p*
Δ Total cholesterol	−0.128	0.162
Δ HDL cholesterol	−0.059	0.648
Δ LDL cholesterol	−0.163	0.203
Δ oxLDL	−0.048	0.727
Δ ApoA1	−0.212	0.135
Δ ApoB	−0.072	0.614
Δ Lp(a)	−0.088	0.533
Δ Triglycerides	0.178	0.164

Δ = changes in variables expressed as % of baseline values

*HDL* high density lipoprotein, *LDL* low density lipoprotein, *oxLDL* oxidized low density lipoprotein, *ApoA1* apolipoprotein A1, *ApoB* apolipoprotein B, *Lp(a)* lipoprotein a, *IMT* intima-media thickness

## Discussion

Our results show that the pro-atherogenic lipid profile characteristic of women with severe obesity and the metabolic syndrome turns into a healthier one after bariatric surgery, with a significant increase in HDL and ApoA1, and a decrease in LDL, oxLDL and ApoB. Except for the decrease in LDL which was of a higher magnitude after RYGB, the overall effects on lipid profile was of a similar magnitude after both surgical techniques. However, we could not demonstrate an association of these changes with the decrease in carotid intima-media thickness.

In humans, adipose tissue stores about a quarter of total body cholesterol whereas in obese individuals this figure is increased up to 50% [28]. Furthermore, a high cholesterol content is also found in muscle, skin and connective tissues with increased body fat [29]. This accumulation of cholesteryl esters is essential for atherosclerotic plaque formation, as well as for the rupture of vulnerable plaque that results into cardiovascular events [28]. Consequently, the important weight loss achieved by bariatric surgery reduces the risk of myocardial infarction, stroke, cardiovascular events and cardiovascular mortality compared to non-surgical controls [30].

Even though not every patient with severe obesity shows what has been called a “metabolically unhealthy obese phenotype” [31], when present, the mixed dyslipidemia associated with obesity is characterized by increased triglycerides, LDL and oxLDL and an increased proportion of smaller and atherogenic LDL particles, as well as reduced HDL concentrations [32, 33]. Dyslipidemic patients with obesity may experience a beneficial change in their lipid profiles after weight loss, including bariatric surgical procedures. It typically reduces triglyceride levels and often increases HDL levels, also with a decrease of triglyceride-rich lipoproteins and a decreased in LDL particle number with a decreased proportion of smaller LDL particles [28].

OxLDL are proatherogenic lipoproteins that are thought to result from the oxidation of small, dense LDL particles [34]. Increased adiposity in obese subjects contributes to low-grade inflammation [35] and global oxidative stress by increasing the production of reactive oxygen species [36]. Conversely, bariatric surgery induces a reduction in inflammation [37] and oxidative stress [36, 38], decreases plasma oxidative markers, including oxLDL and Lp-PLA2 activity [20].

Our present results expand the already published effects of bariatric surgery on circulating lipids by conducting a direct comparison of these outcomes in subjects submitted to different bariatric surgery techniques. In this regard, we here show that SG and RYGB are very similar in terms of eliciting beneficial changes in lipid profiles 1 year after the intervention. Yet, despite occurring in parallel to a reduction of the carotid intima-media thickness in these patients, the beneficial changes in the lipid profile were not associated with the decrease in carotid intima-media thickness already reported [21].

Together with weight loss, there are several possible mechanisms that may contribute to explain why bariatric surgery may reduce CV events apart from the effects on lipids: a reduction of visceral fat, a reduction of white blood cells, and probably more importantly the decreased levels of serum molecules mediating endothelial dysfunction and inflammation [39]. We have recently shown that the increase in adiponectin concentrations achieved after bariatric surgery drives many beneficial metabolic changes but shows no association with the decrease in carotid intima-media thickness [40]. It is also possible that, in the short-term, the amelioration of inflammation could be a more relevant factor in decreasing the cardiovascular risk than other beneficial metabolic changes - including lipid changes - but that on the contrary, in the long term, the resolution of diabetes, hypertension and dyslipidemia might be more relevant. Furthermore, the effects of bariatric surgery on glucagon like peptide 1 (GLP-1) concentrations, which have been shown to be of a different magnitude after RYGB and SG [41], may also explain in part the differences between these two surgical techniques on lipid profiles. Future long-term studies should address these issues.

Our study has several advantages such as being based on a homogeneous population in terms of age, baseline cardiovascular risk and a strict follow up of patients submitted to obesity surgery. Besides, by including only women, we avoided the possible interference of sexual dimorphism on metabolic function and cardiovascular risk [42]. However, the lack of randomization in the allocation of the patients to the different interventions represents a major limitation. Non-random allocation biased adiposity and use of statins towards higher magnitudes in the subgroup of patients submitted to RYGB. Another limitation of our study is that sample size analysis was performed to find differences in carotid intima-media thickness and not in lipid profiles, so we cannot exclude the possibility of a higher efficacy of RYGB than sleeve. In fact, the proportion of patients who could stop statins was higher after RYGB than after sleeve, and this is also a positive effect of the former.

Two recent studies have compared the effects on lipid profiles after RYGB and SG: Heffron and coworkers showed a similar weight loss after these two surgical techniques but a higher increase in HDL and ApoA after SG [43]; on the other hand, Maraninchi and coworkers also showed a similar weight loss after these two surgical techniques, with similar increases in HDL and reductions in triglycerides, but they showed a higher decrease in total cholesterol, LDL and ApoB after RYGB [44]. Although the effects of bariatric surgery on lipid profiles are clearly beneficial, small differences between surgical techniques need further studies due to small discrepancies which may be due to different selected populations and sample size of studies.

## Conclusions

In conclusion, both RYGB and SG induce a significant increase in HDL and ApoA1, and a decrease in LDL, oxLDL and ApoB, in women with severe obesity and a high cardiovascular risk defined by the presence of the metabolic syndrome.

## Abbreviations

ANOVA: Analysis of variance
ApoA1: Apolipoprotein A1
ApoB: Apolipoprotein B
BMI: Body mass index
CV: Coefficient of variation
EBW: Excess body weight
ELISA: Enzyme link immunosorbent assay
EWL: Excess weight loss
GLM: General linear model
GLP-1: Glucagon like peptide 1
HDL: High density-lipoprotein
LDL: Low density-lipoprotein
Lp(a): Lipoprotein a
OxLDL: Oxidised low density-lipoproteins
RYGB: Roux-en-Y gastric bypass
SCORE: Systematic Coronary Risk Evaluation
SG: Sleeve gastrectomy
WC: Waist circumference

## References

[1] Finucane MM, Stevens GA, Cowan MJ, Danaei G, Lin JK, Paciorek CJ, Singh GM, Gutierrez HR, Lu Y, Bahalim AN, Farzadfar F, Riley LM, Ezzati M (2011). National, regional, and global trends in body-mass index since 1980: systematic analysis of health examination surveys and epidemiological studies with 960 country-years and 9.1 million participants. Lancet.

[2] Berrington de Gonzalez A, Hartge P, Cerhan JR, Flint AJ, Hannan L, MacInnis RJ, Moore SC, Tobias GS, Anton-Culver H, Freeman LB, Beeson WL, Clipp SL, English DR, Folsom AR, Freedman DM, Giles G, Hakansson N, Henderson KD, Hoffman-Bolton J, Hoppin JA, Koenig KL, Lee IM, Linet MS, Park Y, Pocobelli G, Schatzkin A, Sesso HD, Weiderpass E, Willcox BJ, Wolk A, Zeleniuch-Jacquotte A, Willett WC, Thun MJ (2010). Body-mass index and mortality among 1.46 million white adults. N Engl J Med.

[3] Yumuk V, Tsigos C, Fried M, Schindler K, Busetto L, Micic D, Toplak H (2015). European guidelines for obesity management in adults. Obes Facts.

[4] Poirier P, Giles TD, Bray GA, Hong Y, Stern JS, Pi-Sunyer FX, Eckel RH (2006). Obesity and cardiovascular disease: pathophysiology, evaluation, and effect of weight loss: an update of the 1997 American Heart Association scientific statement on obesity and heart disease from the obesity Committee of the Council on nutrition, physical activity, and metabolism. Circulation.

[5] Whitlock G, Lewington S, Sherliker P, Clarke R, Emberson J, Halsey J, Qizilbash N, Collins R, Peto R (2009). Body-mass index and cause-specific mortality in 900 000 adults: collaborative analyses of 57 prospective studies. Lancet.

[6] Buchwald H, Oien DM (2013). Metabolic/bariatric surgery worldwide 2011. Obes Surg.

[7] Balsa JA, Botella-Carretero JI, Gomez-Martin JM, Peromingo R, Arrieta F, Santiuste C, Zamarron I, Vazquez C (2011). Copper and zinc serum levels after derivative bariatric surgery: differences between Roux-en-Y Gastric bypass and biliopancreatic diversion. Obes Surg.

[8] Balsa JA, Botella-Carretero JI, Peromingo R, Caballero C, Munoz-Malo T, Villafruela JJ, Arrieta F, Zamarron I, Vazquez C (2010). Chronic increase of bone turnover markers after biliopancreatic diversion is related to secondary hyperparathyroidism and weight loss. Relation with bone mineral density. Obes Surg.

[9] Chapin BL, LeMar HJ, Knodel DH, Carter PL (1996). Secondary hyperparathyroidism following biliopancreatic diversion. Arch Surg.

[10] Coates PS, Fernstrom JD, Fernstrom MH, Schauer PR, Greenspan SL (2004). Gastric bypass surgery for morbid obesity leads to an increase in bone turnover and a decrease in bone mass. J Clin Endocrinol Metab.

[11] Busetto L, Dicker D, Azran C, Batterham RL, Farpour-Lambert N, Fried M, Hjelmesaeth J, Kinzl J, Leitner DR, Makaronidis JM, Schindler K, Toplak H, Yumuk V (2017). Practical recommendations of the obesity management task force of the European Association for the Study of obesity for the post-bariatric surgery medical management. Obes Facts.

[12] Mechanick JI, Youdim A, Jones DB, Garvey WT, Hurley DL, McMahon MM, Heinberg LJ, Kushner R, Adams TD, Shikora S, Dixon JB, Brethauer S (2013). Clinical practice guidelines for the perioperative nutritional, metabolic, and nonsurgical support of the bariatric surgery patient--2013 update: cosponsored by American Association of Clinical Endocrinologists, the Obesity Society, and American Society for Metabolic & Bariatric Surgery. Obesity (Silver Spring).

[13] Yip S, Plank LD, Murphy R (2013). Gastric bypass and sleeve gastrectomy for type 2 diabetes: a systematic review and meta-analysis of outcomes. Obes Surg.

[14] Botella-Carretero JI, Balsa JA, Gomez-Martin JM, Peromingo R, Huerta L, Carrasco M, Arrieta F, Zamarron I, Martin-Hidalgo A, Vazquez C (2013). Circulating free testosterone in obese men after bariatric surgery increases in parallel with insulin sensitivity. J Endocrinol Investig.

[15] Calderon B, Galdon A, Calanas A, Peromingo R, Galindo J, Garcia-Moreno F, Rodriguez-Velasco G, Martin-Hidalgo A, Vazquez C, Escobar-Morreale HF, Botella-Carretero JI (2014). Effects of bariatric surgery on male obesity-associated secondary hypogonadism: comparison of laparoscopic gastric bypass with restrictive procedures. Obes Surg.

[16] Escobar-Morreale HF, Botella-Carretero JI, Alvarez-Blasco F, Sancho J, San Millan JL (2005). The polycystic ovary syndrome associated with morbid obesity may resolve after weight loss induced by bariatric surgery. J Clin Endocrinol Metab.

[17] Sjostrom L (2008). Bariatric surgery and reduction in morbidity and mortality: experiences from the SOS study. Int J Obes.

[18] Sjostrom L, Narbro K, Sjostrom CD, Karason K, Larsson B, Wedel H, Lystig T, Sullivan M, Bouchard C, Carlsson B, Bengtsson C, Dahlgren S, Gummesson A, Jacobson P, Karlsson J, Lindroos AK, Lonroth H, Naslund I, Olbers T, Stenlof K, Torgerson J, Agren G, Carlsson LM (2007). Effects of bariatric surgery on mortality in Swedish obese subjects. N Engl J Med.

[19] Mantyselka P, Kautiainen H, Saltevo J, Wurtz P, Soininen P, Kangas AJ, Ala-Korpela M, Vanhala M (2012). Weight change and lipoprotein particle concentration and particle size: a cohort study with 6.5-year follow-up. Atherosclerosis.

[20] Julve J, Pardina E, Perez-Cuellar M, Ferrer R, Rossell J, Baena-Fustegueras JA, Fort JM, Lecube A, Blanco-Vaca F, Sanchez-Quesada JL, Peinado-Onsurbe J (2014). Bariatric surgery in morbidly obese patients improves the atherogenic qualitative properties of the plasma lipoproteins. Atherosclerosis.

[21] Gomez-Martin JM, Aracil E, Galindo J, Escobar-Morreale HF, Balsa JA, Botella-Carretero JI (2017). Improvement in cardiovascular risk in women after bariatric surgery as measured by carotid intima-media thickness: comparison of sleeve gastrectomy versus gastric bypass. Surg Obes Relat Dis.

[22] Alberti KG, Eckel RH, Grundy SM, Zimmet PZ, Cleeman JI, Donato KA, Fruchart JC, James WP, Loria CM, Smith SC (2009). Harmonizing the metabolic syndrome: a joint interim statement of the international diabetes federation task force on epidemiology and prevention; National Heart, Lung, and Blood Institute; American Heart Association; world heart federation; international atherosclerosis society; and International Association for the Study of obesity. Circulation.

[23] Barroso LC, Muro EC, Herrera ND, Ochoa GF, Hueros JI, Buitrago F (2010). Performance of the Framingham and SCORE cardiovascular risk prediction functions in a non-diabetic population of a Spanish health care Centre: a validation study. Scand J Prim Health Care.

[24] Classification and Diagnosis of Diabetes (2018). Standards of medical care in diabetes-2018. Diabetes Care.

[25] Whelton PK, Carey RM, Aronow WS, Casey DE, Collins KJ, Dennison Himmelfarb C, DePalma SM, Gidding S, Jamerson KA, Jones DW, MacLaughlin EJ, Muntner P, Ovbiagele B, Smith SC, Spencer CC, Stafford RS, Taler SJ, Thomas RJ, Williams KA, Williamson JD, Wright JT (2018). 2017 ACC/AHA/AAPA/ABC/ACPM/AGS/APhA/ASH/ASPC/NMA/PCNA guideline for the prevention, detection, evaluation, and Management of High Blood Pressure in adults: A Report of the American College of Cardiology/American Heart Association Task Force on Clinical Practice Guidelines. J Am Coll Cardiol.

[26] Shah B, Sucher K, Hollenbeck CB (2006). Comparison of ideal body weight equations and published height-weight tables with body mass index tables for healthy adults in the United States. Nutr Clin Pract.

[27] Montero PN, Stefanidis D, Norton HJ, Gersin K, Kuwada T (2011). Reported excess weight loss after bariatric surgery could vary significantly depending on calculation method: a plea for standardization. Surg Obes Relat Dis.

[28] Bays HE, Jones PH, Jacobson TA, Cohen DE, Orringer CE, Kothari S, Azagury DE, Morton J, Nguyen NT, Westman EC, Horn DB, Scinta W, Primack C (2016). Lipids and bariatric procedures part 1 of 2: scientific statement from the National Lipid Association, American Society for Metabolic and Bariatric Surgery, and obesity medicine association: FULL REPORT. J Clin Lipidol.

[29] Crouse JR, Grundy SM, Ahrens EH (1972). Cholesterol distribution in the bulk tissues of man: variation with age. J Clin Invest.

[30] Kwok CS, Pradhan A, Khan MA, Anderson SG, Keavney BD, Myint PK, Mamas MA, Loke YK (2014). Bariatric surgery and its impact on cardiovascular disease and mortality: a systematic review and meta-analysis. Int J Cardiol.

[31] Vazquez C, Arrieta F, Pinera MJ, Balsa J, Martinez-Botas J, Gomez-Coronado D, Calanas A, Zamarron I, Botella Carretero JI (2014). The metabolically unhealthy obese phenotype is mainly associated with hypoadiponectinemia, hyperuricemia and high OPG/RANKL ratio. Clin Nutr ESPEN.

[32] Bays HE, Toth PP, Kris-Etherton PM, Abate N, Aronne LJ, Brown WV, Gonzalez-Campoy JM, Jones SR, Kumar R, La Forge R, Samuel VT (2013). Obesity, adiposity, and dyslipidemia: a consensus statement from the National Lipid Association. J Clin Lipidol.

[33] Botella-Carretero JI, Alvarez-Blasco F, Villafruela JJ, Balsa JA, Vazquez C, Escobar-Morreale HF (2007). Vitamin D deficiency is associated with the metabolic syndrome in morbid obesity. Clin Nutr.

[34] Ishigaki Y, Oka Y, Katagiri H (2009). Circulating oxidized LDL: a biomarker and a pathogenic factor. Curr Opin Lipidol.

[35] Escobar-Morreale HF, Villuendas G, Botella-Carretero JI, Sancho J, San Millan JL (2003). Obesity, and not insulin resistance, is the major determinant of serum inflammatory cardiovascular risk markers in pre-menopausal women. Diabetologia.

[36] Murri M, Garcia-Fuentes E, Garcia-Almeida JM, Garrido-Sanchez L, Mayas MD, Bernal R, Tinahones FJ (2010). Changes in oxidative stress and insulin resistance in morbidly obese patients after bariatric surgery. Obes Surg.

[37] Botella-Carretero JI, Alvarez-Blasco F, Martinez-Garcia MA, Luque-Ramirez M, San Millan JL, Escobar-Morreale HF (2007). The decrease in serum IL-18 levels after bariatric surgery in morbidly obese women is a time-dependent event. Obes Surg.

[38] Sledzinski T, Goyke E, Smolenski RT, Sledzinski Z, Swierczynski J (2009). Decrease in serum protein carbonyl groups concentration and maintained hyperhomocysteinemia in patients undergoing bariatric surgery. Obes Surg.

[39] Boido A, Ceriani V, Cetta F, Lombardi F, Pontiroli AE (2015). Bariatric surgery and prevention of cardiovascular events and mortality in morbid obesity: mechanisms of action and choice of surgery. Nutr Metab Cardiovasc Dis.

[40] Gomez-Martin JM, Balsa JA, Aracil E, Insenser M, Priego P, Escobar-Morreale HF, Botella-Carretero JI. Circulating adiponectin increases in obese women after sleeve gastrectomy or gastric bypass driving beneficial metabolic changes but with no relationship with carotid intima-media thickness. Clin Nutr. 2017; in press 10.1016/j.clnu.2017.10.003 29054470

[41] Casajoana A, Pujol J, Garcia A, Elvira J, Virgili N, de Oca FJ, Duran X, Fernandez-Veledo S, Vendrell J, Vilarrasa N (2017). Predictive value of gut peptides in T2D remission: randomized controlled trial comparing metabolic gastric bypass, sleeve gastrectomy and greater curvature plication. Obes Surg.

[42] Escobar-Morreale HF, Santacruz E, Luque-Ramirez M, Botella Carretero JI (2017). Prevalence of ‘obesity-associated gonadal dysfunction’ in severely obese men and women and its resolution after bariatric surgery: a systematic review and meta-analysis. Hum Reprod Update.

[43] Heffron SP, Lin BX, Parikh M, Scolaro B, Adelman SJ, Collins HL, Berger JS, Fisher EA (2018). Changes in high-density lipoprotein cholesterol efflux capacity after bariatric surgery are procedure dependent. Arterioscler Thromb Vasc Biol.

[44] Maraninchi M, Padilla N, Beliard S, Berthet B, Nogueira JP, Dupont-Roussel J, Mancini J, Begu-Le Corroller A, Dubois N, Grangeot R, Mattei C, Monclar M, Calabrese A, Guerin C, Desmarchelier C, Nicolay A, Xiao C, Borel P, Lewis GF, Valero R (2017). Impact of bariatric surgery on apolipoprotein C-III levels and lipoprotein distribution in obese human subjects. J Clin Lipidol.

